# Evaluation of parents’ attitudes and perceptions regarding the use of artificial intelligence in dental practice: a cross-sectional survey

**DOI:** 10.1186/s12903-026-09320-4

**Published:** 2026-07-20

**Authors:** Esra Hato, Büşra Çelik Kalaağası, Merve Erkmen Almaz, Volkan Arıkan

**Affiliations:** Department of Pediatric Dentistry, Faculty of Dentistry, Kırıkkale University, Kırıkkale, 71450 Türkiye

**Keywords:** Artificial intelligence acceptance, Dental anxiety, Digital literacy, Parents, Perceptions, Questionnaires

## Abstract

**Aim:**

This study aimed to evaluate the attitudes and perceptions of parents of pediatric patients in our sample towards the growing use of artificial intelligence in dental practices.

**Materials and methods:**

The descriptive cross-sectional survey study was conducted with parents of pediatric patients who visited the Kırıkkale University School of Dentistry for routine dental examinations between 2024 and 2025. A 23-question survey was designed for participants to complete. The survey was evaluated as a scale consisting of three sub-dimensions. The data were analyzed using SPSS 27.

**Results:**

A total of 74.4% of participants reported having an average level of self-reported knowledge about the digital world (internet/technology). The biggest advantage of AI identified by the participants was saving time (64.3%). The AI-Health Education Score differed significantly according to educational status (*p* = 0.003). The participants with a bachelor’s degree had higher mean scores on AI-Health Education than high school graduates. The effect size was small (η²=0.051). The mean Physician-AI Collaboration Score for men was significantly higher than that for women. The effect size was small (Cohen’s d = -0.305).

**Conclusion:**

These findings suggest that, within this sample, parents generally view AI positively and may be receptive to its integration into dental practice. These results may support the appropriate integration of AI into dental practice. It is also necessary to assess the effects on patient satisfaction and the quality of healthcare services.

**Supplementary Information:**

The online version contains supplementary material available at https://doi.org/10.1186/s12903-026-09320-4.

## Introduction

 The term “artificial intelligence” (AI) was coined in the 1950s to describe the process of training a computer to think like humans [1, 2]. Today, AI is having an increasing impact on various aspects of daily life. Its impact is evident in fields such as education, healthcare, agriculture, entertainment, communications, engineering, psychology, and politics [3, 4].

The rapid development of AI systems supported by machines and deep learning is paving the way for new healthcare applications. The accumulation of Big Data in healthcare services, in particular, is accelerating the adoption of these systems [5]. These technologies are well-known for their use in the healthcare sector for early diagnosis, accurate identification, clinical decision-making, and health maintenance [6].

As of now, AI is used for various purposes in all branches of dentistry. Reported applications include automated radiograph interpretation, image analysis, tooth detection and segmentation, dental caries and periapical pathology detection, tooth root morphology evaluation, anatomical landmark identification in cephalometric radiographs, abnormality identification in images that cannot be detected through visual interpretation, orthodontic treatment planning, root canal length determination, and digital smile design [7]. To date, various AI models have been trained on image analysis in the field of pedodontics to detect and classify supernumerary teeth, mesiodens, early childhood caries, dentition stage, pit and fissure sealants, dental caries, dental plaque, and ectopic eruptions [8, 9].

Using AI technologies in healthcare services can reduce the burden on professionals, improve care for patients, and compensate the shortages of human resources in the healthcare sector [10]. Assistance of AI is available for clinical diagnosis, treatment planning, scheduling appointments, managing insurance and paperwork, and many other tasks. AI helps dentists to stay informed about their patients’ medical and dental histories and habits [11].

However, despite AI’s successful implementation in dentistry, there are still issues that need to be resolved before it can be used routinely. Although AI technologies have advantages, critical ethical, legal, and social issues must also be addressed. Issues such as the protection of personal data, accountability, and uncertainty regarding liability in the event of potential medical errors affect people’s trust in medical AI [5]. Therefore, the healthy development and widespread adoption of AI technology in healthcare depends on public acceptance of these systems and the integration of experts capable of implementing these innovations.

In addition to the potential benefits of using artificial intelligence (AI) technologies in various fields, it is important that those affected by these technologies understand and accept their use, and that the factors influencing this understanding are investigated. In this context, the Technology Acceptance Model (TAM) developed by Davis [12] provides a widely used framework for understanding technology acceptance behaviors. According to the Technology Acceptance Model, perceived usefulness and perceived ease of use are the main factors that determine users’ attitudes towards technology, and these attitudes influence their intention to use it. The Physician-AI Collaboration score, AI-Health Education score, and AI Errors-Security Concerns score evaluated in this study correspond to the Perceived Usefulness, Attitudes Towards Innovation, and Perceived Risk constructs of the Technology Acceptance Model, respectively.

Children’s oral health is affected by their parents’ knowledge of dental health, attitudes, cultural beliefs, oral hygiene habits, regular dental checkups, care of primary teeth, and concerns about oral health. Parents’ attitudes toward dental care can influence their children’s oral health behaviors and treatment decisions. Therefore, interventions that target parents’ beliefs and practices regarding oral health may be beneficial in preventing oral health problems. Dental treatment for young children is usually provided only after an explanation is given to the parents and their approval is received [13]. In this context, we believe that understanding parents’ general attitudes towards AI systems is a necessary first step, since these systems are not yet routinely used in dentistry. These perceptions will impact parents’ acceptance of AI-supported applications in pediatric dentistry. After determining parents’ perspectives on the use of AI in dentistry, future studies could evaluate their acceptance of specific AI-based applications directly affecting their children.

To our knowledge, no previous studies have examined parents’ attitudes and perceptions regarding the use of artificial intelligence in dentistry. In this context, it is crucial to evaluate how parents perceive the application of AI in dentistry. The adoption and integration of advanced technologies such as artificial intelligence are influenced by societal and cultural contexts [14]. Accordingly, in this study, we aimed to evaluate Turkish parents’ attitudes and perceptions of the use of AI applications in dentistry. Key outcomes included Turkish parents who participated in our study, overall perceptions of AI in dentistry, and their associations with dental anxiety–related factors, education, age, gender, and digital literacy.

## Materials and methods

### Ethical considerations

This study was approved by the Non-Interventional Research Ethics Committee at Kırıkkale University (Decision No. 20240624) and was conducted in accordance with the Declaration of Helsinki.

### Sample size estimation and study participants

The sample size was calculated based on estimating a proportion in a cross-sectional survey. Assuming a 95% confidence level (Z = 1.96), a margin of error of 5%, and an expected population proportion of 0.5, the minimum required sample size was calculated as 384 participants. Eq. was used for the sample size calculation [15]:

$$\:n=\frac{{z}^{2}p\left(1-p\right)}{{d}^{2}}$$

where n is the required sample size, p is the expected population proportion, Z is the Z statistic corresponding to the desired confidence level, and d is the margin of error.

The parent-based descriptive cross-sectional survey was conducted with parents of pediatric patients who visited the Kırıkkale University School of Dentistry for routine dental examinations between 2024 and 2025. The parents who voluntarily agreed to participate in the study were mentally healthy and able to complete and understand the questionnaire were included in the sample. Those who declined to complete the questionnaire, lacked digital literacy, or were unable to complete or understand it were excluded. A total of 386 participants who responded to the data collection tool were included in the study.

### Data collection and measurements

The survey items were adapted from previously published questionnaires reported in the literature [16, 17]. Before being included in the study, all items were reviewed by three expert dentists for relevance, clarity, and content validity. To ensure inter-expert agreement, a ‘consensus’ method was adopted; only items agreed upon by all experts were included in the final scale to ensure content validity. The survey had three sections. The first section contained questions about demographic information. The second section contained questions about oral health and digital literacy. The third section of the survey, the Parents’ Artificial Intelligence in Dentistry Perception Scale (Table 1), contained questions that evaluated the parents’ perceptions of AI in dentistry; these items were rated on a 3-point Likert scale 1 = disagree, 2 = undecided, 3 = agree. For items 11–12, the evaluation was as follows: 3 = disagree, 2 = undecided, 1 = agree. In our study, a 3-point Likert scale was used to reduce completion time, participant fatigue, and indecision between options. According to Jacoby and Matell [18], a 3-point Likert scale is sufficient to maintain reliability and validity while facilitating participant responses.

**Table 1:** The third section of the survey: attitudes and perceptions toward artificial intelligence in dental practice

	No, I disagree.	I’m undecided.	Yes, I agree.
Physician-AI Collaboration			
1. I believe artificial intelligence applications can effectively diagnose diseases.	1	2	3
2. I believe AI applications can provide accurate diagnoses.	1	2	3
3. I believe that artificial intelligence can help prevent anxiety and fear that often arise during dental examinations.	1	2	3
4. I believe that doctors and artificial intelligence can work together.	1	2	3
5. I believe collaboration between doctors and artificial intelligence could make me trust them more.	1	2	3
6. I believe that artificial intelligence can correct errors that may occur due to a dentist’s mistakes.	1	2	3
AI-Health Education			
7. I believe artificial intelligence will advance the fields of medicine and dentistry.	1	2	3
8. I believe that artificial intelligence should be incorporated into medical and dental education.	1	2	3
9. I think humans are more likely to make mistakes than computers are.	1	2	3
AI Errors-Security Concerns			
10. I believe artificial intelligence will revolutionize the fields of medicine and dentistry.	1	2	3
11. I’m worried that artificial intelligence might cause errors, leading to incorrect decisions by dentists. .	3	2	1
12. I am concerned about data security and privacy issues related to artificial intelligence.	3	2	1

The Parents’ Artificial Intelligence in Dentistry Perception Scale consisted of 12 items and comprised the following three subdimensions: Physician–AI Collaboration (6 items), AI–Health Education (3 items), and AI Errors–Security Concerns (3 items). Higher scores indicated more positive perceptions towards the use of AI in dental practice. The psychometric properties of the scale were evaluated using exploratory factor analysis and reliability analysis. The analysis revealed a KMO value of 0.714. The Bartlett’s sphericity test results were significant (χ² = 1324.558; df = 66; *p* < 0.001). These results suggest that the dataset is suitable for factor analysis. Exploratory factor analysis revealed that the items formed a three-factor structure, accounting for 56.88% of the total variance. Physician-AI Collaboration explained 25.52% of the total variance, AI-Health Education Score accounted for 18.83%, and AI Errors-Security Concerns contributed 12.53%. Cronbach’s alpha values for the sub-dimensions of the scale were 0.742, 0.770, and 0.432, respectively, and 0.647 for the total scale. Detailed statistical results are provided in Appendix 1.

The data collection process was managed by a single investigator. The questionnaire was administered as a self-reported online survey using Google Forms. The survey link was distributed digitally, and the participants completed the questionnaire independently without any investigator involvement during completion. The collected data were automatically recorded by the platform and exported by the same investigator for subsequent analysis. Consent was obtained by asking, “Do you agree to participate in the survey and fill out the survey?” as the first question of the survey. To ensure privacy and confidentiality, individual results were not disclosed.

### Data analysis and evaluation

The data were analyzed using the SPSS 27 program. Exploratory factor analysis was applied to determine the factor structure, and confirmatory factor analysis was performed using the AMOS-22 program to verify the goodness-of-fit values of the obtained factors. A frequency table was created for sociodemographic questions. Kolmogorov-Smirnov analysis was used to examine the normality of the data distribution. This revealed that neither the scale nor its sub-dimensions exhibited a normal distribution (*p* < 0.05). As the kurtosis and skewness values of the scale and its sub-dimensions did not exceed the − 2 to + 2 limit, parametric tests were employed in the subsequent analyses, with attention also paid to the homogeneity of variances. Parametric tests were used for both the scale and its sub-dimensions. Furthermore, non-parametric tests were used for sociodemographic questions when the number of observations was insufficient (*n* < 30), even if the scale and its sub-dimensions were normally distributed. To examine differences in group means, an independent samples t-test and a nonparametric Mann-Whitney U analysis were performed for two-group variables, while a one-way ANOVA and a nonparametric Kruskal-Wallis H analysis were performed for three or more group variables. Pearson’s correlation analysis was performed to determine the strength and direction of the relationship between the scales. The analyses also included a Bonferroni correction to reduce Type I error at the α = 0.05 level. To manage the risk of Type I error in multiple tests, a Bonferroni correction was utilized for the comparison group, which included the three subscales and the total scale. This adjustment led to a revised alpha level of 0.0125 (0.05 divided by 4).

## Results

A total of 386 parents—191 women and 195 men—participated in our study. Most participants were aged between 36 and 50 years old and had a bachelor’s degree. The participants’ self-reported knowledge about AI is given in Table 2.

**Table 2:** Distribution of participants according to answers to questions

	Group	*n*	(%)
Part 1: Demographic Information			
2. Your gender	Female	191	49.5
	Male	195	50.5
3. Your age	18–25	62	16.1
	26–35	75	19.4
	36–50	186	48.2
	51–65	62	16.1
	65 and above	1	0.3
4. Your education level	None	0	0.0
	Primary school	6	1.6
	High school	66	17.1
	Bachelor’s degree	259	67.1
	Master’s degree	49	12.7
	Doctorate/PhD	6	1.6
Part 2: Oral Health And Digital Literacy			
5. How important is your oral health to you?	Not important	4	1.0
	Slightly important	4	1.0
	Moderately important	71	18.4
	Very important	307	79.5
6. Does the amount of time the dentist spends making a diagnosis affect your dental anxiety level?	Yes	250	64.8
	No	80	20.7
	I don’t know	56	14.5
7. Are you satisfied with the speed of dental treatments?	Yes	178	46.1
	No	158	40.9
	I don’t know	50	13.0
8. What do you think about your level of knowledge about the digital world (internet/technology)?	I have no knowledge	12	3.1
	I have below average knowledge	42	10.9
	I have average knowledge	287	74.4
	I have above average knowledge	45	11.7
9. Have you heard of the term artificial intelligence?	Yes	374	96.9
	No	12	3.1
10. How would you evaluate your level of knowledge about artificial intelligence?	I have no knowledge	15	3.9
	I have below average knowledge	119	30.8
	I have average knowledge	241	62.4
	I have above average knowledge	11	2.8
11. In your opinion, what is the biggest advantage of artificial intelligence? (More than one option can be selected.)	Saving time	249	64.3
	Better doctor-patient relationships	99	25.6
	More accurate diagnosis	185	47.8
	Increased treatment quality	183	47.3
	Improved understanding of health care	193	49.9
	Greater compliance with standards among dentists	118	30.5
	Other	38	9.8

Furthermore, 64.8% of the participants reported that the amount of time the dentist spends making a diagnosis affected their level of dental anxiety. When asked, “Are you satisfied with the speed of dental treatments?”, 46.1% of the participants answered “yes,” while 40.9% answered “no.”

Overall, 74.4% of the participants reported having an average level of knowledge about the digital world (internet/technology). A statistically significant relationship was found between gender and level of knowledge about the digital world (internet/technology) (*p* = 0.009, X^2^: 11.449). Among the participants who reported having no knowledge about the digital world, females were predominant, whereas among those who reported having above-average knowledge, males were predominant.

Nearly all participants (96.9%) stated that they had heard the term AI. More specifically, 62.4% of the participants reported having an average level of knowledge about AI, while 30.8% reported having below-average knowledge. No statistically significant relationship was found between gender and self-reported knowledge level regarding artificial intelligence (*p* = 0.692, X^2^: 1.457).

The biggest advantages of AI as seen by the participants were saving time, improved understanding of health, more accurate diagnosis, increased treatment quality, greater compliance with standards among dentists, and better doctor-patient relationships.

No statistically significant differences were found in the scores for Physician–AI Collaboration, AI-Health Education, and AI Errors–Security Concerns based on variables such as having heard of the term AI, age, satisfaction with the speed of treatment and knowledge of the digital world (*p* > 0.0125).

According to the level of knowledge about artificial intelligence variable groups, there were no statistically significant differences in the Physician-AI Collaboration Score, The AI-Health Education Score and the Scale Total Score (*p* > 0.0125). The AI Errors-Security Concerns Score significantly differed according to the level of knowledge about artificial intelligence (*p* = 0.008). The participants with no knowledge of artificial intelligence had higher mean scores on the AI Errors-Security Concerns Score than participants with above-average knowledge of artificial intelligence. The effect size was small (η² = 0.044).

The Physician-AI Collaboration Score significantly differed between gender groups (*p* = 0.003) (Table 3). The mean Physician-AI Collaboration Score for males was significantly higher than the mean score for females. The effect size was small (Cohen’s d = -0.305). However, the AI-Health Education Score, AI Errors-Security Concerns Score, and Total Scale Score did not show statistically significant differences according to gender.

**Table 3:** Comparison of AI scale scores by gender

Scale	Group	*n*	Mean	SD	t	df	*p*	Levene (*p*)	Cohen’s d
Physician-AI Collaboration	Female	191	13.26	2.790	-2.994	384	0.003*	0.272	− 0.305
	Male	195	14.09	2.660					
AI-Health Education Score	Female	191	6.60	2.049	0.726	384	0.468	0.225	0.074
	Male	195	6.45	2.168					
AI Errors-Security Concerns	Female	191	5.77	1.447	1.355	384	0.176	0.078	0.138
	Male	195	5.58	1.307					
Total Scale	Female	191	25.92	3.731	− 0.732	384	0.465	0.823	− 0.075
	Male	195	26.19	3.585					

*SD* Standard Deviation, *df* Degrees of freedom

Bonferroni correction results in 0.05/4 = 0.0125 *p < 0.0125

Table 4 presents the results of the one-way ANOVA analysis for the variable “effect of diagnosis time on dental anxiety level.” There was a statistically significant difference in participants’ Physician–AI Collaboration scores based on their perceptions of the impact of diagnosis time on dental anxiety (*p*=0.000), and the effect size was small (η² = 0.043). Participants who believed that diagnosis time had no effect on dental anxiety had significantly higher Physician–AI Collaboration scores than those who believed it did have an effect and those who were undecided (or unaware) regarding its effect. Furthermore, the Physician–AI Collaboration scores of participants who believed that the diagnosis time affected dental anxiety were significantly higher than those of participants who were unaware of this effect.

**Table 4:** Effect of diagnosis time on dental anxiety level variable one-way ANOVA analysis

Scale	Group	*n*	Mean	SD	df	F	*p*	Levene (*p*)	Eta Squared (η²)
Physician-AI Collaboration	a) Yes	250	13.70	2.816	2	8.607	0.000*	0.090	0.043
	b) No	80	14.45	2.311					(a > c)
	c) I don’t know	56	12.50	2.683					(b > a, b > c)
	Total	386	13.68	2.753					
AI-Health Education Score	a) Yes	250	6.62	2.097	2	0.751	0.473	0.636	0.004
	b) No	80	6.36	2.177					(-)
	c) I don’t know	56	6.32	2.072					
	Total	386	6.52	2.109					
AI Errors-Security Concerns	a) Yes	250	5.58	1.336	2	2.163	0.116	0.070	0.011
	b) No	80	5.73	1.242					(-)
	c) I don’t know	56	6.00	1.695					
	Total	386	5.67	1.380					
Total Scale	a) Yes	250	26.18	3.582	2	4.089	0.018	0.384	0.021
	b) No	80	26.54	3.493					(-)
	c) I don’t know	56	24.82	4.000					
	Total	386	26.06	3.656					

*SD* Standard Deviation, *df* Degrees of freedom

Bonferroni correction results in 0.05/4 = 0.0125 *p < 0.0125

Table 5 shows the results of the Kruskal-Wallis H analysis for the educational status variable. According to the educational status variable groups, there were no statistically significant differences in the Physician-AI Collaboration Score, the AI Errors-Security Concerns Score and the Scale Total Score (*p* = 0.203, *p* = 0.054 and *p* = 0.045, respectively). The AI-Health Education Score significantly differed according to educational status (*p* = 0.003). The participants with a bachelor’s degree had higher mean scores on AI-Health Education than high school graduates. The effect size was small (η² = 0.051).

**Table 5:** Comparison of AI scale scores by educational status

Scale	Group	*n*	Mean Rank	df	H	*p*	Eta Squared (η²)
Physician-AI Collaboration	a) Primary school	6	145.67	5	5.947	0.203	0.009
	b) High school	66	170.07				(-)
	c) Bachelor’s degree	259	202.17				
	d) Master’s degree	49	185.42				
	e) Doctorate/PhD	6	190.92				
	Total	386					
AI-Health Education Score	a) Primary school	6	126.25	5	15.891	0.003*	0.051
	b) High school	66	150.95				(c > b)
	c) Bachelor’s degree	259	204.83				
	d) Master’s degree	49	193.77				
	e) Doctorate/PhD	6	237.42				
	Total	386					
AI Errors-Security Concerns	a) Primary school	6	295.25	5	9.289	0.054	0.020
	b) High school	66	199.53				(-)
	c) Bachelor’s degree	259	191.05				
	d) Master’s degree	49	195.48				
	e) Doctorate/PhD	6	115.08				
	Total	386					
Total Scale	a) Primary school	6	133.67	5	9.749	0.045	0.027
	b) High school	66	161.70				(-)
	c) Bachelor’s degree	259	204.20				
	d) Master’s degree	49	188.90				
	e) Doctorate/PhD	6	178.75				
	Total	386					

*df* Degrees of freedom

Bonferroni correction results in 0.05/4 = 0.0125 *p < 0.0125

According to the results of correlation analysis, there was a positive and very weak statistically significant relationship between the Physician-AI Collaboration Score and the AI-Health Education Score (*r* = 0.145; *p* = 0.004). Furthermore, we found a positive and strong statistically significant relationship between the Physician-AI Collaboration Score and the Scale Total Score (*r* = 0.681; *p* = 0.000). The results also revealed a positive and strong statistically significant relationship between the AI-Health Education Score and the Scale Total Score (*r* = 0.649; *p* = 0.000). and a positive and weak statistically significant relationship between the AI Errors-Security Concerns Score and the Scale Total Score (*r* = 0.299; *p* = 0.000).

## Discussion

Parents play a crucial role in their children’s growth and health, particularly in the early years. Parental decisions and preferences influence health-related choices and children’s access to healthcare services [19]. It also affects their children’s treatment options and processes. Parents’ trust in AI technologies is closely related to their acceptance of their use in pediatric dentistry.

Based on the self-reported information provided by parents who participated in our study, it can be concluded that they have a positive view of this issue. These findings are particularly significant, as parental acceptance is likely to play a crucial role in the successful integration of AI-based applications into pediatric dental practice. Integrating developing AI technology into diagnostic and treatment processes can help parents to better understand complex procedures and facilitate better communication with pediatric dentists.

It is widely known that every field is greatly affected by AI today. As evidenced by the increasing number of studies on AI applications in dentistry, AI is increasingly influencing the field of dentistry as well [20–22]. Considering the constant changes in technology, it is important to evaluate dental patients’ perceptions, opinions, and concerns regarding the use of AI in dentistry. Accordingly, in this study, we aimed to evaluate the opinions of parents, as legal guardians of their children, about the use of AI in dentistry.

The results showed that 64.8% of participants believed that the diagnosis time affected dental anxiety, while half of the participants were satisfied with the speed of treatment, and the other half were not. A significant difference was found between diagnosis time and Physician-AI Collaboration scores, but no significant relationship was found with treatment speed. This may indicate that parents are particularly sensitive to uncertainty and the diagnosis time, especially during the diagnostic phase. Accordingly, the use of AI in dentistry can be considered to be not solely based on expectations of speed, but may be more related to perceptions of control, trust, and transparency. One possible explanation is that AI-powered systems may increase perceptions of predictability and transparency during diagnosis. Supporting this interpretation, previous studies reported that AI-based diagnostic systems help patients to better understand, believe in, and trust dentists’ radiographic diagnoses [17, 23]. According to Milgrom et al. [24], one of the significant sources of dental phobia is a lack of trust in dental staff; therefore, this approach can help to reduce dental anxiety.

Furthermore, according to Günec et al., 55.7% of the participants reported that the length of the physician’s diagnosis affected their level of dental anxiety [16]. Similarly, in a study conducted by Sharka et al., the participants reported perceiving more benefits and fewer risks associated with the adoption of AI in dental treatment, indicating a generally positive attitude towards its application in clinical practice [25]. Therefore, the participants’ perceptions of AI as a reliable, supportive, and useful technology that works in harmony with dentists may reduce dental anxiety linked to distrust of dentists. Further studies are needed to clarify how different diagnostic durations affect anxiety levels.

In our results, 74.4% of participants had an average level of self-reported knowledge about the digital world, specifically the internet and technology. Statistically significant differences in knowledge of the digital world were observed depending on gender. Among the participants who reported having no knowledge about the digital world, females were predominant; the reverse trend was observed among male participants. Furthermore, the mean Physician-AI Collaboration Score for men was higher than the mean score for women. These results suggest that men have a more positive attitude towards AI in dental practices and are more likely to trust it. It should be noted, however, that this finding reflects perceived attitudes, rather than inherent gender-based tendencies, and that although statistically significant, the observed effect sizes were small, suggesting limited practical significance. Indeed, a previous study found that a larger percentage of females reported having basic knowledge of AI (78.4%) as compared to males (72.6%), and women were slightly more aware of its use in dentistry [26]. In addition, previous research documented no statistically significant differences based on gender [26]. Similarly, a study in Saudi Arabia revealed no significant difference between male and female participants in terms of their willingness to accept AI in dental treatment [25].

Cultural factors influence attitudes toward the use of artificial intelligence in dentistry. Furthermore, in a study on patient acceptability for the use of AI in dental radiology in India, Arora et al. reported that age, education, and residential background were significantly related to patients’ awareness and perception of AI [27]. Cultural influences are not explicitly examined in our study. International research is needed to better understand how sociocultural factors shape the use of artificial intelligence in dentistry.

In contrast to the findings of Arora et al. [27], in the present study, no statistically significant association was found between age and perceptions of artificial intelligence. This finding may be attributed to the fact that most participants were concentrated in the 36–50 age group. The uneven distribution of participants across the age groups may have made it difficult to identify the effect of age.

Previous research also documented that participants’ attitudes towards the use of AI in dental diagnosis were significantly related to their level of education [23]. This is thought to be because people with higher levels of education tend to be more exposed to information, current trends, and technology, which makes them more likely to use AI-based tools [23, 27]. On the other hand, Sharka et al. reported no significant difference in willingness to accept AI-based dental treatment based on educational level [25]. In the present study, the AI-Health Education Score significantly differed according to groups of educational status. Specifically, the participants with a bachelor’s degree had a significantly higher mean of AI-Health Education Score than high school graduates. Although statistically significant, the observed effect sizes were small, suggesting limited practical significance. Educational level is particularly relevant when it comes to understanding AI’s role in education. The lack of differences in safety and error concerns may indicate that these concerns are more widespread and independent of educational level.

Furthermore, Ayad et al. reported that the participants perceived the three most important advantages of using AI in dentistry as “improved diagnostic reliability” (60.8%), “time reduction” (48.3%), and “more personalized and evidence-based disease management” (43.0%) [17]. Similarly, in our study, the participants cited time savings, an improved understanding of healthcare, and more accurate diagnoses as the biggest advantages of AI. These participants evaluated AI primarily in terms of its technical and rational benefits. This perception aligns with evidence from a recent systematic review, which highlighted that AI is widely regarded as an auxiliary tool and that clinical expertise remains indispensable [28]. Based on these results, it can be concluded that patients and parents currently view AI as a supportive tool.

While many patients have positive views on the use of AI in dental practice, some are cautious about AI applications. For instance, in a recent study on patients’ perceptions of the use of robotics and AI in dentistry, Al-Dabbagh found that the participants who were unwilling to undergo treatment performed by a robot alone were most concerned about trust and safety issues [29].

Müller et al. investigated the barriers and enablers to the application of artificial intelligence through a survey of dentists and patients in Germany [30]. The study reported that providing patients with an AI-generated second opinion, alongside color-coded pathological findings in X-ray images, helped them better understand their condition, thereby increasing their confidence in the dentist’s diagnosis. Patients view AI-powered clinics as more contemporary, cutting-edge, and aligned with current standards, according to the study [30].

The literature emphasizes that ultimate responsibility should lie with the dentist [1, 30, 31]. Müller et al. identified data protection breaches, legal uncertainties, and concerns among physicians about losing their clinical skills or critical perspectives due to over-reliance on AI as barriers to the adoption of technology [30]. This concern is referred to as ‘automation bias’ in the literature [32]. This bias manifests in two types of errors: omission errors and commission errors. It has been stated that omission errors occur when people fail to notice or ignore AI errors, while commission errors occur when healthcare professionals mistakenly implement or accept machine decisions despite evidence to the contrary [32]. Agarwal et al. have stated that respondents expressed a strong desire to know when AI is used for their child’s imaging, to be informed how accurate the technology is before its use, and to be asked for permission before AI is used to interpret their child’s scans [33]. This finding highlights the importance of transparency in clinical processes.

Attitudes and perceptions towards AI technology can be affected by factors such as time, cultural background, and individual differences, as well as perceived technological efficiency, perceived risk of job loss, level of trust, and subjective norms [33, 34]. A recent systematic review revealed significant variability in dental patients’ knowledge and awareness levels about AI, even among studies conducted in the same countries and over similar time periods [28]. This variation was attributed to the highly subjective and context-dependent nature of the information reported by the patients. Furthermore, the review highlighted the lack of valid tools to objectively evaluate patients’ knowledge of AI [28].

This study has several limitations. First, the collection of data based on the parents’ self-reported responses may have led to response bias. It may not accurately reflect actual knowledge or understanding of AI technologies. Second, the use of a 3-point Likert scale may have limited the sensitivity of participant responses. The third limitation is the distribution of the survey via an online link, which may have led to selection bias and coverage bias by preventing the inclusion of parents with low digital literacy. Therefore, this could affect the generalizability of our results for the entire population. Since the questionnaire was distributed online, parents who lacked digital literacy could not be included in the sample. This may have resulted in selection and coverage bias and reduced the variance of the variables under investigation. Therefore, the lack of significant differences between groups regarding awareness of the term ‘artificial intelligence’, satisfaction with the speed of treatment, and knowledge of the digital world should be interpreted in light of this caveat. Future large-scale studies that include disadvantaged groups lacking digital literacy are anticipated to highlight the differences between groups with a higher effect size. Additionally, the fact that the data collection phase is carried out by a single researcher can also be considered as a researcher bias. Fourth, the study was conducted in a single center and a single geographic region, which limits external validity and generalizability of the findings to other regions or healthcare settings. To increase the generalizability of the results, in further research, multicenter studies covering different regions and including larger sample groups should be conducted. Fifth, to avoid discouraging the participants by a lengthy questionnaire, the number of survey questions was limited. Accordingly, in the future, more comprehensive studies could involve questioning patients and parents in greater detail about which dental treatment procedures they prefer to use with AI support and about which procedures they have reservations in terms of AI use. Another limitation of our study is the low Cronbach’s alpha value for ‘AI Errors-Security Concerns’. The third subscale demonstrated relatively low internal consistency (Cronbach’s α = 0.432), suggesting that the items may represent a heterogeneous construct. Therefore, findings related to this subscale should be interpreted cautiously.

## Conclusion

These findings suggest that, within this sample, parents generally view AI positively and may be receptive to its integration into dental practice. These results may support the appropriate integration of AI into dental practice. It is also necessary to assess the effects on patient satisfaction and the quality of healthcare services. Enhancing digital literacy and addressing ethical and safety-related concerns may improve acceptance and confidence in AI-based dental technologies. As AI continues to be integrated into dental practice, understanding parental perceptions will be essential for guiding patient-centered communication strategies and supporting the responsible and effective future use of AI in dentistry.

## Supplementary Information

Supplementary Material 1.

## Data Availability

The datasets used and/or analyzed in this study are available from the corresponding author on reasonable request.

## References

[1] Schwendicke F, Samek W, Krois J. Artificial intelligence in dentistry: chances and challenges. J Dent Res. 2020;99:769–74. 10.1177/0022034520915714. PMC7309354 32315260

[2] Kong S-C, Cheung WM-Y, Zhang G. Evaluating artificial intelligence literacy courses for fostering conceptual learning, literacy and empowerment in university students: Refocusing to conceptual building. Comput Hum Behav Rep. 2022;7:100223. 10.1016/j.chbr.2022.100223.

[3] Laupichler MC, Aster A, Haverkamp N, Raupach T. Development of the Scale for the assessment of non-experts’ AI literacy–An exploratory factor analysis. Comput Hum Behav Rep. 2023;12:100338. 10.1016/j.chbr.2023.100338.

[4] Wang B, Rau P-LP, Yuan T. Measuring user competence in using artificial intelligence: validity and reliability of artificial intelligence literacy scale. Behav Inf Technol. 2023;42:1324–37. 10.1080/0144929X.2022.2072768.

[5] Uygun İlikhan S, Ozer M, Tanberkan H, Bozkurt V. How to mitigate the risks of deployment of artificial intelligence in medicine? Turk J Med Sci. 2024;54:483–92. 10.55730/1300-0144.5814. PMC11265878 39050000

[6] Yilmaz Y, Yilmaz DU, Yildirim D, Korhan EA, Kaya DÖ. Artificial intelligence and the use of artificial intelligence in health: opinions of health sciences students. Suleyman Demirel Univ J Health Sci. 2021;12:297–308. 10.22312/sdusbed.950372.

[7] Akdogan C, Ozdemir H. Benefits of artificial intelligence to dental practice. J Turk Dent Res. 2024;2:278–87. 10.58711/turkishjdentres.vi.1296215.

[8] Samaranayake L, Tuygunov N, Schwendicke F, Osathanon T, Khurshid Z, Boymuradov SA, et al. The transformative role of artificial intelligence in dentistry: A comprehensive overview. Part 1: fundamentals of AI, and its contemporary applications in dentistry. Int Dent J. 2025;75:383–96. 10.1016/j.identj.2025.02.005. PMC11976540 40074616

[9] Yuce F, Tassoker M. The applications of artificial intelligence in dentistry. 7tepe Klin Derg. 2023;19:141–9. 10.5505/yeditepe.2023.05668.

[10] Gao S, He L, Chen Y, Li D, Lai K. Public perception of artificial intelligence in medical care: content analysis of social media. J Med Internet Res. 2020;22:e16649. 10.2196/16649. PMC7385634 32673231

[11] Agrawal P, Nikhade P, Nikhade PP. Artificial intelligence in dentistry: past, present, and future. Cureus. 2022;14:e27405. 10.7759/cureus.27405. PMC9418762 36046326

[12] Davis FD. Perceived usefulness, perceived ease of use, and user acceptance of information technology. MIS Q. 1989;13:319–40.

[13] Al-Batayneh OB, Al-Khateeb HO, Ibrahim WM, Khader YS. Parental knowledge and acceptance of different treatment options for primary teeth provided by dental practitioners. Front Public Health. 2019;7:322. 10.3389/fpubh.2019.00322. PMC6854019 31788466

[14] Hegde S, Nanayakkara S, Jordan A, Jeha O, Patel U, Luu V, et al. Attitudes and perceptions of australian dentists and dental students towards applications of artificial intelligence in dentistry: a survey. Eur J Dent Educ. 2025;29:9–18. 10.1111/eje.13042. PMC11729985 39340812

[15] Daniel WW, Cross CL. Biostatistics: a foundation for analysis in the health sciences. 11th ed. Wiley; 2018.

[16] Gunec HG, Gokyay SS, Kaya E, Aydın KC. Is the society ready for dental diagnosis with artificial intelligence? Selcuk Dent J. 2022;9:200–7. 10.15311/selcukdentj.915522.

[17] Ayad N, Schwendicke F, Krois J, Van Den Bosch S, Bergé S, Bohner L, et al. Patients’ perspectives on the use of artificial intelligence in dentistry: a regional survey. Head Face Med. 2023;19:23. 10.1186/s13005-023-00368-z. PMC10288769 37349791

[18] Jacoby J, Matell MS. Three-point likert scales are good enough. J Mark Res. 1971;8:495–500. 10.1177/002224377100800414.

[19] Jafarzadeh M, Ghasemi R, Asnaashari E, Ponnet K, Moeeni M. Your right to choose: parents’ preferences toward a pediatric dental center. BMC Oral Health. 2025;25:386. 10.1186/s12903-025-05707-x. PMC11910009 40089706

[20] Manjulika T, Shailesh J, Maitreyi R, Sahba H, Nikhil P, Kumar D, et al. Artificial intelligence tools in dentistry: A systematic review on their application and outcomes. Cureus. 2025;17:e85062. 10.7759/cureus.85062. PMC12206247 40585609

[21] Lu W, Yu X, Li Y, Cao Y, Chen Y, Hua F. Artificial intelligence–related dental research: bibliometric and altmetric analysis. Int Dent J. 2025;75:166–75. 10.1016/j.identj.2024.08.004. PMC11806303 39266401

[22] Shirani M. Trends and classification of artificial intelligence models utilized in dentistry: A bibliometric study. Cureus. 2025;17:e81836. 10.7759/cureus.81836. PMC12057650 40337568

[23] Bahadir HS, Keskin NB, Cakmak ESK, Gunec G, Cesur Aydin K, Peker F. Patients’ attitudes toward artificial intelligence in dentistry and their trust in dentists. Oral Radiol. 2025;41:52–9. 10.1007/s11282-024-00775-1. 39379636

[24] Milgrom P, Weinstein P, Getz T. Treating fearful dental patients: a patient management handbook. 2nd ed. Seattle: University of Washington, Continuing Dental Education; 1995.

[25] Sharka R, Skatawi B, Sayyam G, Abutaleb M, Alshareef M, Alamar M, et al. Predicting artificial intelligence acceptance in dental treatments among patients in Saudi Arabia: A perceived risks and benefits perspective. Oral. 2025;5:28. 10.3390/oral5020028.

[26] Shrateh ON, Al-batat S, Al-Qudimat AR, Ghannam LI, Abuhanoud LJ. Attitudes and perceptions of dental students towards artificial intelligence. BMC Med Educ. 2025;25:1386. 10.1186/s12909-025-07854-9. PMC12512535 41068696

[27] Arora PC, Sandhu KK, Arora A, Gupta A, Waghmare M, Rampal V. Acceptability of artificial intelligence in dental radiology among patients in India: are we ready for this revolution? Oral Radiol. 2025;41:69–77. 10.1007/s11282-024-00777-z. 39384683

[28] da Cunha Lima K, Aguiar HC, Szonyi V, Fernandes MH, Tirapelli C, Grosgogeat B. Patient perception of the use of artificial intelligence in dentistry: a systematic review and single-arm meta-analysis. Digit Dent J. 2026;3:100071. 10.1016/j.ddj.2026.100071.

[29] Al-Dabbagh N, Alnowailaty Y, Abed H, Sharka R, Alhilou A, Almarghlani A. Patients’ perception of using robotics and artificial intelligence in dentistry: A cross-sectional study. Open Dent J. 2024;18:1–9. 10.2174/0118742106339605240918065914.

[30] Müller A, Mertens SM, Göstemeyer G, Krois J, Schwendicke F. Barriers and enablers for artificial intelligence in dental diagnostics: a qualitative study. J Clin Med. 2021;10:1612. 10.3390/jcm10081612. PMC8069285 33920189

[31] Sciarra FM, Caivano G, Cacioppo A, Messina P, Cumbo EM, Di Vita E, et al. Dentistry in the era of artificial intelligence: medical behavior and clinical responsibility. Prosthesis. 2025;7:95. 10.3390/prosthesis7040095.

[32] Abdelwanis M, Alarafati HK, Tammam MMS, Simsekler MCE. Exploring the risks of automation bias in healthcare artificial intelligence applications: A Bowtie analysis. J Saf Sci Resil. 2024;5:460–9. 10.1016/j.jnlssr.2024.06.001.

[33] Agarwal G, Salami RK, Lee L, Martin H, Shantharam L, Thomas K, et al. Parental and carer views on the use of AI in imaging for children: a national survey. Insights Imaging. 2025;16:172. 10.1186/s13244-025-02021-6. PMC12335414 40782183

[34] Gerlich M. Perceptions and acceptance of artificial intelligence: A multi-dimensional study. Soc Sci. 2023;12:502. 10.3390/socsci12090502.

