# Supplementary material for: Evaluation of parents’ attitudes and perceptions regarding the use of artificial intelligence in dental practice: a cross-sectional survey
Source: BMC Oral Health. 2026 Jul 20;26:2015. doi: 10.1186/s12903-026-09320-4 (PMC13662973; doi:10.1186/s12903-026-09320-4)
Supplement: Supplementary file 1 [file 12903_2026_9320_MOESM1_ESM.docx]

***Artificial intelligence scale validity and reliability findings***

When the Scree Plot graph of the Parents' Artificial Intelligence in Dentistry Perception Scale is examined, it is understood that it can be three-dimensional (Figure 1).

The results of the Exploratory Factor Analysis are given in Table 1. When the KMO coefficient and Bartlett's test of sphericity results are examined, it is seen that the scale is suitable for factor analysis. As a result of exploratory factor analysis, it was seen that the items had a structure consisting of 3 factors, explaining 56.88% of the total variance. The first factor explains 25.52% of the total variance, the second factor explains 18.83% of the total variance, and the third factor explains 12.53% of the total variance. As a result of the exploratory factor analysis, items numbered 13, 12, 16, 15, 17 and 14 were gathered under the 1st factor, items numbered 20, 19 and 21 were gathered under the 2nd factor, and items numbered 22, 23 and 18 were gathered under the 3rd factor. Cronbach's alpha values appear to be at a sufficient level based on reliability analyses performed for the three factors and the total score, as well as the total scale score. Factor loadings for the three-factor model are shown in Figure 2.

The results of the Multifactor Model for the Parents' Perception Scale of Artificial Intelligence in Dentistry are presented in Table 2. When correlations between other variables are examined, it is seen that the factor loadings of the items are above 0.30 and all correlations are significant.

The Goodness of Fit Values of the Structural Model are given in Table 3. According to the Confirmatory Factor Analysis, it was determined that the structural equation modeling results of the scale were significant at the p=0.000 level, and the 12 items that make up the scale were related to the 3-sub-dimensional scale structure. The model is being improved. During the improvement, variables that reduce fit were identified, and new covariances were created for those with high covariance among residual values. The accepted values for the fit indices in the subsequent revised fit index calculations are shown in the table.

Table 4 shows normality and reliability analyses. Kolmogorov-Smirnov analysis was used to examine the normality of the data distribution. This revealed that neither the scale nor its sub-dimensions exhibited a normal distribution (p<0.05). As the kurtosis and skewness values of the scale and its sub-dimensions did not exceed the -2 to +2 limit, parametric tests were employed in the subsequent analyses, with attention also paid to the homogeneity of variances. Parametric tests were used for both the scale and its sub-dimensions. Furthermore, non-parametric tests were used for sociodemographic questions when the number of observations was insufficient (n < 30), even if the scale and its sub-dimensions were normally distributed.

**Table 1. Exploratory Factor Analysis Table for Parents' Perception Scale of Artificial Intelligence in Dentistry**

| **Itemler** | **F1** | **F2** | **F3** | **Dimension Reliability** | **Scale Reliability** |
| --- | --- | --- | --- | --- | --- |
| Item 13 | ,795 |  |  | ,742 | ,647 |
| Item 12 | ,770 |  |  |  |  |
| Item 16 | ,571 |  |  |  |  |
| Item 15 | ,545 |  |  |  |  |
| Item 17 | ,375 |  |  |  |  |
| Item 14 | ,353 |  |  |  |  |
| Item 20 |  | ,822 |  | ,770 |  |
| Item 19 |  | ,753 |  |  |  |
| Item 21 |  | ,619 |  |  |  |
| Item 22 |  |  | ,667 | ,432 |  |
| Item 23 |  |  | ,607 |  |  |
| Item 18 |  |  | ,391 |  |  |
| Variance Explained: | %25,52 | %18,83 | %12,53 | Total: %56,88 | |
| KMO: ,714 |  | χ^2^:1324,558 | | Sd:66 | Sig: ,000 |

**Table 2. Results of the Multifactor Model of the Parents' Perception Scale on Artificial Intelligence in Dentistry**

| **Factors** | **Items** | **Factor Load** | **Standard Error** | **t** | **p** |
| --- | --- | --- | --- | --- | --- |
| Factor1 | Item 13 | ,854 |  |  |  |
|  | Item 12 | ,827 | ,068 | 13,260 | *** |
|  | Item 16 | ,492 | ,059 | 9,060 | *** |
|  | Item 15 | ,458 | ,049 | 8,401 | *** |
|  | Item 17 | ,373 | ,031 | 4,951 | *** |
|  | Item 14 | ,324 | ,067 | 5,907 | *** |
| Factor2 | Item 20 | ,857 |  |  | *** |
|  | Item 19 | ,759 | ,074 | 11,207 | *** |
|  | Item 21 | ,577 | ,065 | 9,853 | *** |
| Factor3 | Item 22 | ,666 |  |  | *** |
|  | Item 23 | ,702 | ,204 | 5,957 | *** |
|  | Item 18 | ,310 | ,262 | 4,564 | *** |

***p<0.05

**Table 3. Goodness of Fit Values of the Structural Model**

|  | **Structural Model Values** | **Recommended Values** |
| --- | --- | --- |
| χ^2^/df | 4,051 | ≤ 5 |
| RMSEA | ,089 | ≤0,1 |
| CFI | ,883 | ≥0,80 |
| IFI | ,884 | ≥0,80 |
| TLI | ,842 | ≥0,80 |
| NFI | ,852 | ≥0,80 |
| GFI | ,922 | ≥0,80 |
| SRMR | ,050 | ≤0,10 |
| **χ^2^: 198,499; df:49; p:0,000** | | |

**Table 4. Normality Assumption Analysis and Reliability Analysis**

| **Scale** | **n** | **Mean** | **SD** | **Kolmogorov Smirnov (p)** | **Skewness** | **Kurtosis** | **Cronbach’s Alpha** |
| --- | --- | --- | --- | --- | --- | --- | --- |
| Factor1 | 386 | 13,68 | 2,753 | ,000 | -,961 | ,428 | ,742 |
| Factor2 | 386 | 6,52 | 2,109 | ,000 | -,519 | -1,012 | ,770 |
| Factor3 | 386 | 5,67 | 1,380 | ,000 | ,679 | ,492 | ,432 |
| ÖlçekToplam | 386 | 26,06 | 3,656 | ,000 | -1,143 | 1,941 | ,647 |

*p>0,05

Figure-1: Scree Graph of the Number of Factors in the Parents' Perception Scale of Artificial Intelligence in Dentistry

Figure 2: Multifactorial Model of Parents' Perception Scale of Artificial Intelligence in Dentistry
